# Identification of Barriers to Adherence to a Weight Loss Diet in Women Using the Nominal Group Technique

**DOI:** 10.3390/nu12123750

**Published:** 2020-12-06

**Authors:** Angela De Leon, James N. Roemmich, Shanon L. Casperson

**Affiliations:** Grand Forks Human Nutrition Research Center, U.S. Department of Agriculture, Agricultural Research Service, 2420 2nd Ave. North, Grand Forks, ND 58203, USA; adeleon@ucdavis.edu (A.D.L.); james.roemmich@usda.gov (J.N.R.)

**Keywords:** barriers, diet, nominal group technique, weight loss, women

## Abstract

Background: At any given time, a majority of women are engaged in some type of weight loss diet; however, these efforts are difficult to sustain for long-term weight control. Because women are more likely to develop obesity and suffer a greater severity of obesity-related health and economic consequences, we sought to identify the key factors that make adhering to a weight loss diet difficult for overweight/obese women. Methods: Ten nominal group technique (NGT) sessions aimed at identifying perceived barriers to adherence to a weight loss diet were conducted as part of a weight loss study for overweight/obese women (*n* = 33) during the controlled feeding weight loss phase. Results: Individual-level barriers to emerge from the sessions included knowing when to stop eating, being able to control cravings and emotional eating, and sustaining healthier dietary habits. Environmental-level barriers included family/social events that bring people together, especially those centered around food and drink, eating out, cost, and busy schedules. Conclusions: These findings offer a deeper understanding of barriers women find most salient to adhering to a weight loss diet, providing direction for the clinical application of weight loss programs.

## 1. Introduction

At any given time, many women, especially those of childbearing age, are engaged in some form of weight loss diet [1]. Based on self-report measures, women tend to be more invested in food-related issues, are more likely to perceive themselves as needing to lose weight, and thus, are more prone to go on a weight loss diet than are men [2,3]. Furthermore, it has been shown that women do not work as hard as men to gain access to their most liked energy-dense snack food [4]. These findings belie the fact that while overall obesity rates are not different between women and men, women are more likely to have severe obesity [5] (body mass index (BMI) ≥ 40 kg/m^2^) and to experience a greater severity of obesity-related health problems [6,7]. Correspondingly, the economic burden of being overweight or having obesity is greater in women. Dor and colleagues report that in 2010, the costs for women were almost double that for men ($4879 vs. $2646, respectively), with the difference in costs being mainly related to lost wages [8]. These findings highlight the importance of creating an evidence-based platform for successful weight loss interventions for women.

As part of a study investigating the effects of daily dietary protein distribution on weight loss in women, we conducted a series of nominal group technique (NGT) sessions aimed at identifying and prioritizing perceived barriers to adherence to a weight loss diet. The NGT is a well-established small group meeting technique [9,10,11] prized for its ability to foster creativity and generate a large number of different responses to a specific research question. Originally developed by Van de Ven and Delbecq [9] for exploring priorities in the healthcare sector [12,13], NGT has been used in nutrition research to assess barriers and facilitators to compliance with the Dietary Guidelines for Americans (DGA) [14,15,16]. Utilizing NGT, we sought to identify the key factors that make adhering to a weight loss diet difficult for women. While many of the barriers identified in the current study—emotional eating, cravings, and lack of social support—have been reported previously [17,18,19,20], an advantage of the NGT over other methods (e.g., questionnaires, focus groups) is that participants not only generate but also prioritize responses in accordance with their personal experience. Because the NGT sessions were conducted during the controlled feeding weight loss phase of the primary study, participants were uniquely situated to provide “expert” responses, not just hypothetical imaginings or memory-based responses. This is the first research to use NGT to ascertain the barriers most important for successful weight loss in women during a dietary intervention where the barriers of planning, cost, convenience, and preparation time have been removed.

## 2. Materials and Methods

### 2.1. Participants

Healthy women, aged 20–44 years with a BMI of 28–45 kg/m^2^ and a stable body weight, were recruited from the greater Grand Forks, North Dakota area and surrounding communities. To be eligible for enrollment in the parent study, participants provided their own transportation to the Grand Forks Human Nutrition Research Center (GFHNRC) and agreed to continue their usual physical activity practices. Exclusion criteria included: currently dieting to lose weight, inability or unwillingness to attend group meetings or to follow instructions, more than a 10% change in body weight within the previous 2 months, history of eating or gastrointestinal disorders, previous bariatric surgery, allergies to or unwillingness to eat any study foods, current or planned pregnancy, lactation, untreated/uncontrolled metabolic illness/disease, use of tobacco products, alcohol or drug abuse. This study was conducted in accordance with the Declaration of Helsinki and all procedures involving research study participants were approved by the University of North Dakota Institutional Review Board (IRB-201706-363) and registered with ClinicalTrials.gov (NCT03202069). All participants provided written informed consent and were compensated monetarily for their participation.

### 2.2. Dietary Intervention

The overall objective of the parent study, which was a randomized parallel feeding study, was to test the effect of two patterns of daily protein intake on changes in body composition and dietary adherence during weight loss. Participants were randomized into one of two groups: (1) dietary protein provided in an even distribution pattern (30 g at breakfast, lunch, and dinner) and (2) dietary protein provided in a skewed distribution pattern (10 g at breakfast, 15 g at lunch and 65 g at dinner). The parent study consisted of an 8-week controlled feeding phase followed by an 8-week ad libitum phase in which participants were instructed and counseled on maintaining a similar dietary protein intake pattern when buying, choosing, and consuming their own foods.

Resting metabolic rate (RMR) was measured via indirect calorimetry after an overnight fast and multiplied by an activity factor, based on responses to a validated physical activity questionnaire (Stanford Brief Activity Survey) [21]), to determine energy needs. Total energy intake provided in the study diet was 80% of the participants’ calculated energy needs. Participants received all meals and some beverages (2% milk) packaged as 3 meals per day on a 5-day rotation for 8 weeks. The diets differed between the groups only in the daily protein distribution pattern. Meals were prepared in the GFHNRC Metabolic Kitchen using commercially available ingredients and were packaged separately as breakfast, lunch, and dinner to ensure compliance to study conditions. Participants were instructed to eat only the foods provided by the study. Each meal was to be consumed in a single eating occasion and snacking was prohibited. Participants picked up their food daily Monday through Friday, with weekend foods picked up on Friday.

### 2.3. Nominal Group Technique

The NGT follows a highly structured format that precludes interaction among group members [11]. A single researcher with extensive experience in the NGT served as the facilitator for all NGT sessions and provided in-house training to the recorder for each session. At the start of each NGT session, the facilitator welcomed participants, clarified the overall purpose of the group meeting, and explained how the results would be used. Briefly, steps of the NGT sessions were conducted as follows: (1) silent idea generation during which participants are given 5–7 min to write down as many different ideas as possible in response to the question, “What kinds of things make it hard *for people* to stick to a weight loss diet?”; (2) round-robin sharing of ideas while a recorder writes all responses verbatim on a flipchart; (3) reading aloud and clarifying the meaning of all listed ideas without discussion of merit or importance of the ideas; (4) individual choosing and ranking of the five ideas most relevant to the participant *personally from all group responses*, and finally, after a break in the meeting in which the facilitator and the recorder tabulate the results and create a list of top-scoring responses; (5) top-scoring ideas are written on a new page of the flipchart and participants individually rate the group’s 10 top-scoring ideas on a scale of 0 to 10 with 10 being most important to them personally and 0 being not important at all. Participants were enrolled and started the primary study in cohorts and the first behavioral weight loss class, consisting of the NGT session, occurred in week 5 of the study. Thus, all participants had been in the weight loss phase of the primary study for four weeks when they participated in NGT sessions.

### 2.4. Data Analysis

Results from the NGT sessions are summarized using descriptive statistics as previously described [14]. Responses that had been chosen by multiple group members as one of their top five ideas most relevant to the participant personally were also included in the final rating scoresheets (up to 10 ideas per group). Scores for each idea on the final rating scoresheets from step 5 were summed and divided by the number of participants in each group. The results from all the NGT sessions were analyzed following the convention in the NGT literature which uses a multi-step prioritization and ranking of responses to a research question [22,23]. Verbatim responses of the top scoring ideas, most important to participants personally, from each NGT group were entered into a spreadsheet to generate a master list of top-scoring ideas. One researcher independently examined the top-scoring ideas to identify common themes generated across groups to create a conceptual thematic framework. For example, ideas generated across groups and rated in the top 10 most important to them personally that were similar but worded slightly differently than in another group (e.g., “cravings” vs. “craving sweets”, etc.) these responses and their scores were combined to generate a single thematic grouping. A second researcher reviewed and clarified the initial thematic framework. Researchers then met to discuss the thematic groupings in an iterative process until reaching consensus on the final themes and corresponding responses [22].

## 3. Results

A total of 33 women aged 32.5 ± 8.3 years with a BMI of 35.5 ± 3.9 kg/m^2^ participated in the 10 NGT sessions. The racial breakdown of participants was 85% White, 9% African American, and 6% American Indian. Previous NGT work [14] demonstrated that after 8 NGT sessions no new ideas were presented that changed the overall thematic framework or the relative rankings of themes. However, because some of the NGT groups were smaller than the recommended minimum of 5 participants (smallest group, *n* = 2), 2 additional groups were added after the first 8 NGT sessions to ensure thematic saturation. Reanalysis of themes including responses from the additional two groups confirmed that thematic saturation was achieved.

**Figure 1:**
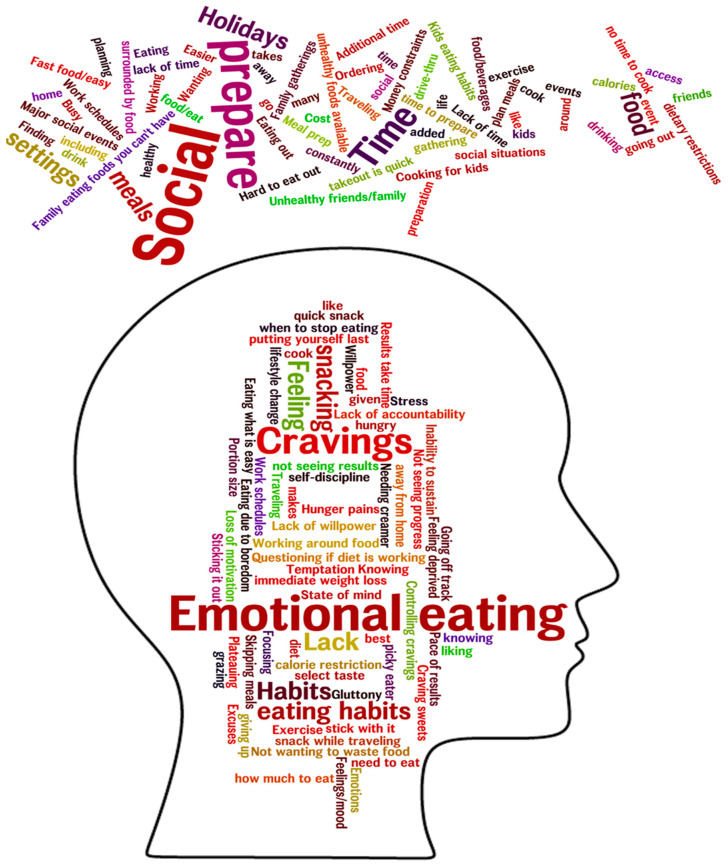
Word cloud of individual-level and environmental-level barriers to adhering to a weight loss diet. Word cloud imagery presented here is a graphical depiction of the number of times barriers within the same theme were repeated across ten nominal group technique (NGT) groups. The more often a barriers response was mentioned, the larger the font size of the word. Words within the head represent individual-level barriers. Words above the head represent environmental-level barriers. The all-directions layout of the environmental barriers represents how ubiquitous they are.

In response to the question, “What sorts of things make it hard for people to stick to a weight loss diet”, the NGT sessions generated between 24 and 48 different ideas per group. Top-scoring barriers, most important to participants *personally*, averaged across all groups, resulted in a number of core themes that could be separated into two main categories classified as individual-level and environmental-level. The top individual-level core themes included (1) knowledge, (2) cravings, (3) emotions, (4) habits, (5) impatience, and (6) willpower. The top environmental-level core themes were (1) family/social, (2) time constraints, (3) eating out, (4) food being present, and (5) cost. Figure 1 uses word cloud imagery to depict the top individual- and environmental-level barriers across groups. Font size indicates the frequency of occurrence of each word/phrase in the aggregated barriers, although some words with smaller font sizes may have had higher overall ratings across groups.

### 3.1. Preceived Individual-Level Barriers

Individual-level barriers related to knowledge included “portion size”, “knowing how much to eat” and “hard to know when to stop eating”. Barriers related to cravings were both in general for snacks and in specific for sweets and included “controlling cravings”. Barriers related to emotions were centered around eating to deal with negative emotions (“emotional eating”), and “stress” eating. Barriers related to habit included “eating due to boredom”, “previous eating habits”, “feeling the need to snack while traveling”, and “skipping meals”. Barriers related to impatience included “feeling like you are not seeing results and giving up”, “pace of results”, and “lack of immediate weight loss”. Barriers related to willpower included “loss of motivation”, “inability to sustain lifestyle change”, “temptation of a quick snack”, “feeling deprived”, and “going off track one day”.

### 3.2. Preceived Environmental-Level Barriers

Environmental-level barriers related to family/social included responses such as “major social events”, “eating is a social event”, “family gatherings”, “holidays”, “wanting to drink with friends”, “unhealthy friends/family (friends/family who eat unhealthy foods)”, and “cooking for kids/kids eating habits”. Barriers related to time included “time for planning and preparation”, “ordering takeout is quick”, “busy schedule”, and “no time to cook”. Barriers related to eating out included “fast food/easy access”, “traveling away from home”, “hard to eat out with dietary restrictions”, and “more unhealthy foods available than healthy (when eating out)”. Barriers related to food being present included “being constantly surrounded by food” and “working around food”. Finally, barriers related to cost were simply stated as “money constraints”, “not wanting to waste food”, and “cost”.

In addition to the environmental-level responses that comprised core themes, several responses were elicited in multiple NGT groups when participants were asked to respond *thinking about people in general*. These included “eating in front of the TV”, “working late means eating late”, “food as a reward or recognition”, “commercials about food on the TV”, “feeling hungry with limited portions”, “unable to ignore junk food (temptation) when (grocery) shopping”, “cultural expectations”, and “convenience”.

## 4. Discussion

This is the first research to use NGT to ascertain the highest rated barriers to adhering to a weight loss diet in women with overweight or obesity while taking part in a controlled feeding weight loss study. Beyond supporting the findings of previous research [19,24], the utilization of the NGT provided a more nuanced understanding of the perceived weight loss barriers women find most important. Both individual-level and environmental-level factors were identified as playing key roles. It is vital that practitioners involved in obesity prevention and treatment understand the barriers women find most salient to adhering to a weight loss diet in order to improve behavioral strategies for successful outcomes.

Starting with the individual-level factors most relevant to this sample of women, it appears there is a disconnect between homeostatic mechanisms of satiety and satiation and current standards of portion sizes. Knowing when to stop eating or how much to eat was rated as one of the greatest barriers to sticking to a weight loss diet. Spence and colleagues [25] conducted a series of focus groups to explore factors influencing consumers’ portion size selection and consumption and reported confusing or inconsistent serving size guidance as a key barrier to appropriate portion size control. In previous generations, people did not need to “know” when to stop eating because proper portion sizes were served in homes and in restaurants. Over the past several decades, however, portion sizes have steadily increased resulting in greater energy intake [26,27]. This powerful phenomenon is posited as a key factor in the rise of obesity rates. Thus, this perceived individual-level barrier to weight loss actually has an environmental-level aspect. Considering that portion sizes are unlikely to shrink back to 1970’s amounts [28], weight loss programs that teach participants to attend to internal hunger and satiety cues through mindfulness and intuitive eating techniques are especially valuable [29].

Cravings were identified as a core barrier; however, not all groups rated it as one of the most important for them personally. Craving is defined as an intense desire for a specific food or taste that can occur in the absence of hunger [26]. While it might seem logical to assume that the sustained dietary energy deficit necessary for weight loss would increase the frequency and strength of food cravings, increases in cravings may have more to do with feelings of deprivation induced by the restriction of “forbidden” foods [26]. During the controlled feeding phase of this study, participants completed daily hunger and satiety questionnaires that also included open-ended questions regarding cravings (data not shown). Responses to questions regarding being distracted by thoughts of food and about the frequency and strength of cravings revealed that cravings occurred most often as a result of being in a situation in which other individuals were observed consuming high-calorie, highly palatable foods or by being in an environment where those foods were readily available. These findings support a stronger association between feelings of deprivation and cravings than between hunger and cravings in this sample of women and highlight the impact of environmental-level factors on triggering cravings in individuals with high food cue reactivity, as documented by Boswell and Kober [30].

Poor eating habits were among the individual-level barriers rated highly by most groups and were mentioned in one way or another by all groups. Whereas habits are simply thought of as behaviors that are repeated over time, van ‘t Riet and colleagues argue that habits are more than just repeated behaviors [31]. They describe the necessary components of habits as “learned sequences of acts that have been reinforced in the past by rewarding experiences and that are triggered by the environment to produce behavior, largely outside of people’s conscious awareness” [31]. Key elements of habits, and why they are so enduring, are that they require no conscious effort or thought, are less strongly guided by intentions, and depend more on situational or environmental cues than intention-based behaviors. Therefore, breaking existing eating habits is unlikely to be successful with educational approaches alone but has been shown to be successful with strategies focused on controlling situational cues, creating contingency plans, and engaging in self-monitoring, such as food logging [32].

Impatience was a barrier that was brought up in all groups, either most important personally or in terms of people in general. Feeling that weight loss should be happening sooner and in greater amounts, questioning whether the diet is working at all, and acknowledging unrealistic expectations were common responses. In the primary study, the amount of energy restriction of the weight loss diet was not severe, and the planned rate of weight loss was 1–2 pounds per week. Thus, it is unsurprising that for participants at the upper end of the BMI range this rate of weight loss might elicit feelings of impatience as they would only be losing a small percentage of their body weight each week. Maintaining willpower/motivation to stick to a weight loss diet in the long term was another major barrier, especially if weight loss was not perceived as happening quickly enough. For women with a high BMI, weight-neutral interventions that emphasize intuitive eating as a main goal to improve health may provide a more viable and satisfying alternative to programs focused solely on weight loss [33].

Finally, emotional eating emerged as a major individual-level barrier to sticking to a weight loss diet. Eating in response to stress or negative emotions, which may be comforting in the short term, could result in obesity in the long term if this becomes a habit. Conversely, having overweight or obesity could cause body image dissatisfaction, increasing psychological stress and depression, potentially triggering emotional eating. A meta-analysis of 15 longitudinal studies found that persons with obesity had a 55% greater risk of developing depression over time, and persons with depression at baseline had a 58% greater risk of becoming obese [34]. One longitudinal study that found a bidirectional relationship between obesity and depression in women, but not in men, also found that emotional eating partially mediated that relationship in both directions [35]. Thus, mindfulness-based interventions (MBIs) aimed at reducing problematic eating behaviors like emotional or stress eating may be beneficial to sustaining a weight loss diet. Research using MBIs has shown that the cultivation of mindfulness can be effective in changing obesity-related eating behaviors, including emotional eating [36,37].

Regarding the environmental-level barriers, responses related to family and social life were considered the most important. Because this was a controlled feeding study, participants were restricted to eating only study foods. This was difficult for those individuals who lived with family/roommates who were eating more desirable foods, especially when the study participant was responsible for preparing the food. Feeling that family and friends were unsupportive of weight loss efforts was identified as a nearly insurmountable barrier. Attending parties, holiday celebrations, and other social events where highly palatable food was present also proved challenging. These responses reflect a feeling of not only being deprived of particular food and drink others are consuming, but perhaps more importantly, a sense of being left out of the group. Research into social modeling of food intake, the phenomenon whereby people adapt their food intake to that of others, suggests that modeling reflects an attempt to affiliate or develop a social bond with one’s eating partners [38,39]. Thus, participants’ negative experiences in social situations while dieting may partly be reflective of their inability to model others’ food intake and thus affiliate with others. These findings underscore the importance of having a supportive social network when undertaking a weight loss diet [18].

Time constraints emerged as another external barrier that was rated highly by all the groups. The perception that sticking to a weight loss diet is more time consuming included not only the time for meal planning and preparation, but also the time it takes to eat, presumably referring to extra time needed to chew fresh foods versus processed foods. Other responses suggest that for some, cooking at home at all, not just for weight loss, is considered a “waste” of time with no associated benefits. Eating out was a barrier that was frequently mentioned and reflected both a concern about the lack of healthy options when eating out and the convenience of fast food versus cooking at home. These barriers are of concern for long term weight management because a greater frequency of food consumption away from home has been associated with greater BMI and lower intake of fruits and vegetables [40]. Foods prepared away from home tend to be energy dense and to be made with greater amounts of fat and sugar than meals prepared at home [40]. Conversely, greater time spent on home food preparation is associated with greater overall diet quality, including greater consumption of fruits and vegetables [41]. Preparing and eating most meals at home would aid in weight control efforts because it places the locus of control over food choices and preparation methods in the hands of the preparer.

Finally, cost was considered a key barrier, which was surprising considering that the NGT sessions occurred during the controlled feeding phase of the study. It is possible that the provision of a healthy diet, conducive to maintaining a healthy body weight, made the participants more aware of the costs of healthy foods. The fact that calorie-for-calorie healthy diets cost more than unhealthy diets is well documented [42,43]. In a review examining the relationship between food prices and diet quality of lower-socioeconomic status groups in multiple countries, Darmon and Drewnowski confirmed that energy-dense foods comprising refined grains, added sugars, and fats are less expensive on a per calorie basis than nutrient-dense foods such as fresh fruits and vegetables [44]. Thus, the foods most likely to contribute to overweight and obesity are more likely to be financially desirable. In addition, if one considers the monetary cost of time spent in meal preparation, cooking, and clean-up in addition to food prices, it is easy to understand why some individuals find sticking to a weight loss diet more costly [45].

A strength of this study was the use of NGT, a widely used small group meeting technique, to answer a specific question regarding perceived barriers to being able to stick to a weight loss diet. The nominal nature of NGT stipulates equal participation from all group members and protects against information loss due to group dynamics whereby dominant group members could potentially influence or stifle other group members’ responses. Other advantages of the NGT compared to other methods are that it is time efficient, easily implemented, and provides a written record of verbatim responses from participants. The current study had some limitations. Volunteer bias is a potential confounding factor as there may be inherent differences between the women who chose to participate in this weight loss study and those who did not. Because the parent study was aimed at studying the effects of dietary protein patterns on weight loss in healthy women of childbearing age, as women in that age category have been shown to be concerned with weight control [1], these results may not be generalizable to older women. Moreover, responses from this sample of predominantly White women may not be representative of responses from women of other racial groups, and the results may not be generalizable to men. However, our findings align with those of previous research [17,18,19] not conducted during a controlled feeding trial. Although the overall number of participants was small, with group sizes ranging from 2 to 5 participants, each group generated between 24 and 48 different responses and responses that received multiple votes were included in the final scoring stage. This ensured that not just the strength or relative importance of a response but also the frequency or popularity of a response was reflected in the overall thematic analysis. Moreover, the small sample size of some of our NGT sessions is not uncommon in this literature, as reviewed by McMillan and colleagues [22].

## 5. Conclusions

These findings highlight the interplay between individual-level (internal) and environmental-level (external) factors that may trigger a woman to behave incongruently with her weight loss intentions. Many of the perceived barriers that are considered internal, such as willpower, were intertwined with external factors, such as environmental cues/triggers, underscoring the complexity of adhering to a weight loss diet. The reported lack of knowledge of appropriate portion sizes, difficulty dealing with emotional and stress eating, and the importance of securing buy-in from family and friends, in addition to other findings of this research, suggest specific areas for further study and provide direction for the clinical application of weight loss programs. For example, focusing on health benefits instead of just weight loss shifts the goal to a more holistic endeavor and may be more sustainable in the long term. Specific instruction aimed at increasing self-efficacy in skills integral to healthy weight maintenance could include menu planning, shopping, and cooking, as well as contingency planning for social situations where food and drink are central.
